# Evolution and forecasting of PM10 concentration at the Port of Gijon (Spain)

**DOI:** 10.1038/s41598-020-68636-5

**Published:** 2020-07-16

**Authors:** Fernando Sánchez Lasheras, Paulino José García Nieto, Esperanza García Gonzalo, Laura Bonavera, Francisco Javier de Cos Juez

**Affiliations:** 1 Department of Mathematics, Faculty of Sciences, University of Oviedo, c/ Federico García Lorca 18, 33007 Oviedo, Spain; 2 Department of Physics, Faculty of Sciences, University of Oviedo, c/ Federico García Lorca 18, 33007 Oviedo, Spain; 3 Department of Mining Exploitation and Prospecting, University of Oviedo, c/ Independencia 13, 33004 Oviedo, Spain

**Keywords:** Environmental impact, Environmental sciences

## Abstract

The name PM_10_ refers to small particles with a diameter of less than 10 microns. The present research analyses different models capable of predicting PM_10_ concentration using the previous values of PM_10_, SO_2_, NO, NO_2_, CO and O_3_ as input variables. The information for model training uses data from January 2010 to December 2017. The models trained were autoregressive integrated moving average (ARIMA), vector autoregressive moving average (VARMA), multilayer perceptron neural networks (MLP), support vector machines as regressor (SVMR) and multivariate adaptive regression splines. Predictions were performed from 1 to 6 months in advance. The performance of the different models was measured in terms of root mean squared errors (RMSE). For forecasting 1 month ahead, the best results were obtained with the help of a SVMR model of six variables that gave a RMSE of 4.2649, but MLP results were very close, with a RMSE value of 4.3402. In the case of forecasts 6 months in advance, the best results correspond to an MLP model of six variables with a RMSE of 6.0873 followed by a SVMR also with six variables that gave an RMSE result of 6.1010. For forecasts both 1 and 6 months ahead, ARIMA outperformed VARMA models.

## Introduction

### The town of Gijón and its Port

Gijón is a town located on the north coast of Spain, in the Principality of Asturias. It is the most populated municipality of this region, with a total of 273,422 inhabitants according to 2016 census. This town, together with Oviedo (220,648 inhabitants) and Avilés (79,514 inhabitants) and other small towns, forms a metropolitan area with more than 850,000 inhabitants. It was founded in the fifth century B.C. During the twentieth century it underwent significant development due to industry, something which is still of great importance to the local economy.

The weather in Gijón is defined by its proximity to the sea and the low mean altitude. The annual level of precipitation is quite high, with a total of 920 L per square meter and year. Regarding temperature, the coldest month is January, with an average temperature of 8.9 °C, while the hottest is August with 19.7 °C. The average annual temperature is 13.8 °C. Winds are sporadic and seasonal. The wind regime is dominated by two main components^1^. During winter it blows from W-WSW, while in summer it comes from E-ENE on the coast.

**Figure 1:**
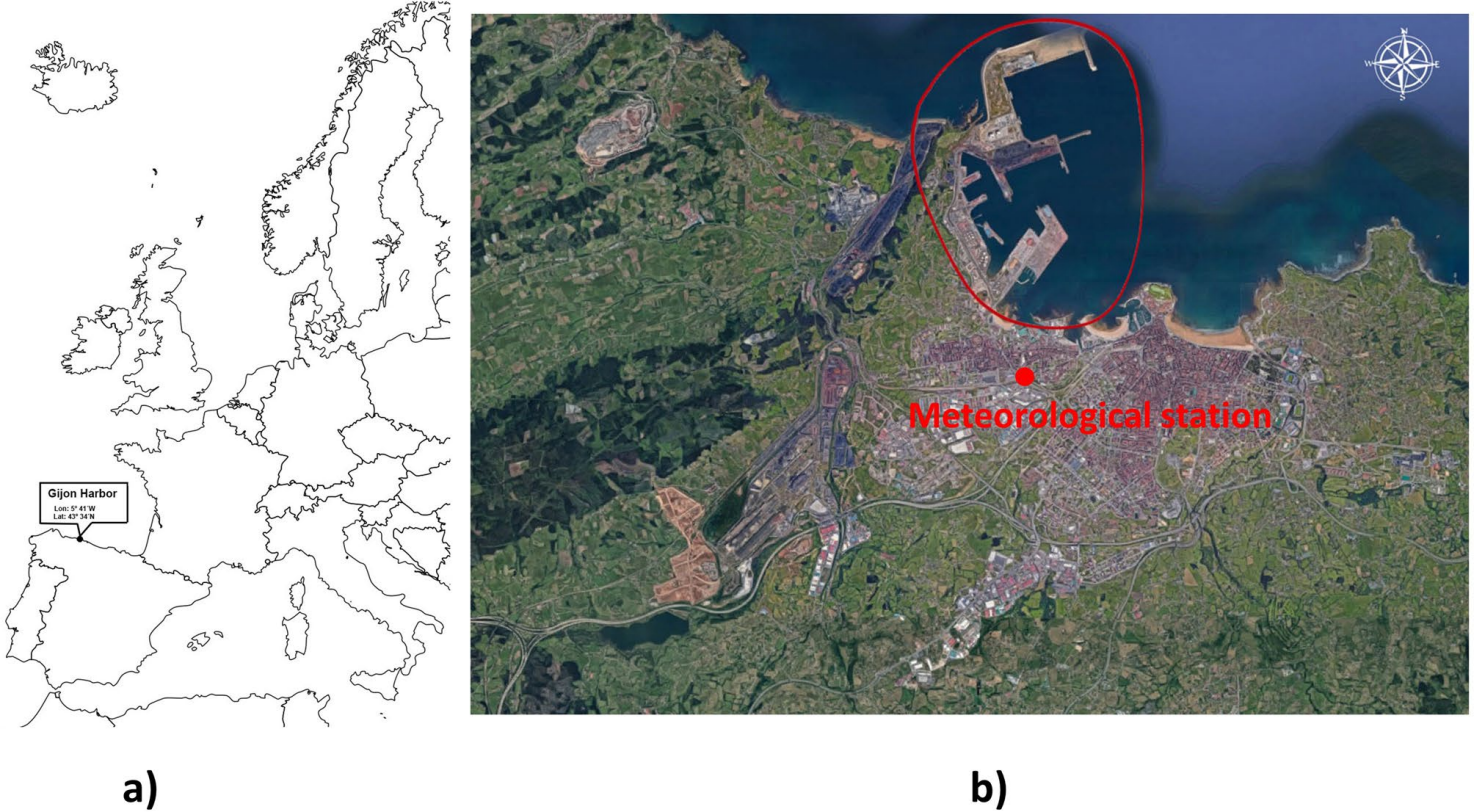
(**a**) position of the Port of Gijon on the North Atlantic coast of Spain, (**b**) aerial picture of Gijón and its Port (inside the red line) including the position of the weather station. *Source*: Google Maps, Map data©2019 Google; https://www.google.es/maps/@43.5547854,-5.6995551,9849m/data=!3m1!1e3. The map was edited with PowerPoint version: 16.0.12527.20260.

The Port of Gijón, named *El Musel*, is one of the main ports of the Atlantic Arc and the leading port in the movement of solid bulk in Spain. It is located in the Cantabrian Sea (43°34′N, 5°41′W). Figure 1a shows its position on the North Atlantic Spanish coast and Fig. 1b is an aerial picture of the town, where the location of the port can be observed.

The commercial exploitation of this port started in 1907. In the 1990s there was a development plan that doubled its area and which led to a significant increase in its activity. Its infrastructure is adapted to modern market requirements in terms of drafts, springs and storage areas and a range of services with the best standards of quality. It has 415 hectares of land surface and 7,000 linear meters of dock, structured in areas with the appropriate characteristics to serve each kind of traffic, i.e. specialized terminals for solid bulks, liquids and containers, and multi-purpose facilities for various types of traffic.

In the beginning, the exports were mainly iron ore and coal. Subsequently, the port would expand on its breakwaters and piers, and in the 1940s became the main Spanish port in traffic movement. The industrial activity of the Principality of Asturias has its main ally in the Port of Gijón. Currently, it is the main bulk port in Spain and one of the most important ports of the Atlantic Arc. According to the traffic statistics of the Annual Report of 2018, a total of 18,226 ships entered the port during that year, which meant a total of 79,294 containers and 12.7 millions of tonnes in the dry bulk terminal, of which 6.4 corresponded to iron ore, 3.4 to iron steel and 2.8 to steam coal. The net revenue in 2018 was 42.2 million euros.

### Pollution and particulate matter studies

The World Health Organisation has reported that air pollution has an adverse effect on people’s health and development^2^. It is well-known that long-term exposure to high levels of air pollution is linked to decrements in lung function in children^3^. A Swiss study found increased levels of allergic sensitisation in adults living in proximity to busy roads for periods longer than 10 years^4^. Also, the PM_10_ pollutant is amongst those regulated under the Air Quality Framework Directive on ambient air quality assessment and management^5^.

A continuous exposure to pollutants such as Carbon Monoxide (CO), Carbon Dioxide (CO_2_), oxides of nitrogen (NO_x_), and particulate matter is reported to cause health problems in the population living in the affected areas^6,7^. Particulate matter is formed by different chemical products, mostly produced by anthropogenic processes^6^ and with significantly variable diameters. Their anthropogenic origin is the reason why they are more present in urban areas^8^ than in unpopulated areas.

Air quality issues are relevant in ports and areas nearby. In general, the duty cycle of marine vessels is longer than that of roadside vehicles. This means that ship engines generally use older technology than cars and due to their engine power they are also much more pollutant^9^. Previous studies have analysed PM_10_ concentrations in ports and coastal areas like the Bay of Algeciras in Southern Spain^10^. Another study analysed the impact of PM_2.5_ particles from ship emissions in southern California^11^. In Turkey, shipping emissions in the regions of Candelari Gulf^12^ and Ambarli Port^13^, both with heavy shipping traffic, were investigated. Research carried out in the port^14^ of Tarragona, Spain, made use of multi-linear regression models to study the contribution of different harbour activities to the levels of particulate matter in its area. In the same line there is another study,^15^performed in Barcelona’s harbour, also located in Spain and about 80 kms. as the crow flies from Tarragona, which has estimated that around 50–55% of PM_10_ and PM_2.5_ concentrations measured at the port could be attributed to harbour activities and that such activities provide about 9–12% of the total PM_10_ concentration in the air and about 11–15% of PM_2.5_ to the metropolitan area of this city Another interesting and innovative study^16^ that deals with the problem of particulate matter in ports was performed in the port of Zhejiang. In this research, with the help of an unmanned aerial vehicle that integrated different sensors, authors have been able to create a profile of the vertical distribution of PM_2.5_, PM_10_ and total suspended particles from ground level to a height of 120 m. A study made at the port of Volos^17^, in Greece, found that the highest PM_10_ concentration values were associated with days of calm winds, meaning a wind speed under $0.5\;\frac{m.}{{s.}}$. The only research into ports that made use of a supervised learning methodology was the one concerning the port of Koper ^18^. Koper is the only port in Slovenia and is located at the northern tip of the Adriatic Sea. Researchers made use of hourly PM_10_ concentrations and employed k-means clustering with Euclidean and city-block distances to cluster days. The results obtained showed the influence of rain intensity and wind speed in the clusters performed but the influence of any other pollutant was not studied. Finally, another study of interest was performed at the Port of Cork, which, like Gijón, is located on the Atlantic coast^19^.

### Use of machine learning techniques to forecast pollutant concentrations

In general, machine learning can be understood as a subset of methodologies of the artificial intelligence field that are able to learn in an automatic way. In other words, they can learn from data and predict future events. Nowadays, the use of machine learning methodologies has extended to almost all branches of science, including environmental studies. One of the main reasons for the use of machine learning approaches for air quality forecasting is the ability of these methodologies to capture non-linear relationships among variables.

Interest in the forecasting of air pollution in urban area dates back to more than a century ago, when large cities began to have problems with pollution ^20^. In the 1970s, several statistical models for pollution forecasting were proposed^21,22^. The first applications of machine learning methodologies in this field were in the 1990s. In those days most research performed made use of artificial neural networks^23,24^.

Since then, the different studies performed have made use of other techniques such as genetic algorithms^25^, Hierarchical Agglomerative Clustering^26^, k-means^27^ or support vector machines as regressors^28^.

Genetic algorithms have been employed as a supporting methodology for selecting the input variables and designing the high-level architecture of neural networks models. In certain research works^25^, they were applied to the selection of the architecture and input variables of a multilayer-perceptron model for forecasting of hourly concentrations of NO. One of the limitations found by this technique is that training each neural network model is a time-consuming task and therefore, the number of parameters to be tuned must be limited.

Hierarchical agglomerative clustering is employed to group objects that are similar in subsets called clusters. The agglomerative clustering methodology starts with many small clusters and merges them together to create large ones. It has been successfully applied in order to study ozone exposure and cardiovascular-related mortality in Canada^26^. The results obtained showed that this methodology is useful for studying the long-term effects of air pollution on cardiovascular diseases.

A recent study has shown how k-means clustering can be employed to categorize different locations in a big and populated city representing the variability of pollution according to the variables employed for the study^27^. Finally, the use of support vector machines as a regressor has also been reported in some studies^28,29^. In one of these^28^ the support vector machine is employed as a regressor model for the forecast of the daily Beijing air quality index from 1^st^ January 2014 to mid-2016, while in the others^29,30^ they are employed for the forecast of the daily average ${PM}_{10}$.

The aim of the present research is to forecast the air quality in a port area, specifically in the port area of the city of Gijón. For this purpose, the article applied different machine learning models (multilayer perceptron neural networks, support vector machine as regressor and MARS) and compared the performance of the predictions obtained for different time intervals with those given by two time series methodologies, one of them univariate (ARIMA) and the other multivariate (VARMA). This means that an exhaustive comparison is made of the prediction from 1 to 6 months in advance of the performance of five methods. This provides an interesting framework for the comparison of methodologies. All these methods were employed in the past for pollution forecasting, but never all in the same research, as far as the authors know. Therefore, the relevance of the present research is that it deals with the topic of monitoring air quality in a city, comparing different machine learning methodologies applied to the same data set.

### The database

The information employed for this research has been obtained from one of the meteorological stations belonging to the network of Air Quality Monitoring of the Government of the Principality of Asturias, and more specifically from the one closest to the Port of Gijón, which is located at Argentina Avenue. This station records environmental measurements hourly. As is normal in all this kind of databases, about 0.23% of the raw observations taken each 15 min for all variables were missing. They were imputed with the help of the Multivariate Imputation by Chained Equiations (MICE) algorithm^31^.

Table 1 shows the minimum, mean, maximum and standard deviation of the pollutants measured at Gijón Port for the period of study. The values considered for the present research were average monthly measurements from January 2010 to June 2018. Information from January 2010 to December 2017 was employed to forecast values from January to June 2018. Pollutants measured at the Port of Gijón were SO_2_, NO, NO_2_, CO, O_3_ and PM_10_.

**Table 1:** Port of Gijón. Minimum, mean, maximum and standard deviation of the variables of the study: sulfur dioxide (SO_2_), nitrogen monoxide (NO), nitrogen dioxide (NO_2_), carbon oxide (CO), ozone (O_3_) and particulate matter with a diameter less than 10 µm (PM_10_).

	Minimum	Mean	Maximum	Standard deviation
SO_2_ (µg/m^3^)	4.0000	7.9706	20.0000	3.2379
NO (µg/m^3^)	4.0000	10.9510	30.0000	6.8091
NO_2_ (µg/m^3^)	7.0000	26.1471	46.0000	8.9159
CO (µg/m^3^)	0.1800	0.4023	0.8600	0.1362
O_3_ (µg/m^3^)	13.0000	38.0000	64.0000	9.9980
PM_10_ (µg/m^3^)	18.0000	31.5196	50.0000	7.6271

## Materials and methods

The present research calculates predictive models of PM_10_ concentration by means of autoregressive integrated moving average (ARIMA), vector autoregressive moving-average (VARMA), multilayer perceptron neural networks (MLP), support vector machines as regressor (SVMR) and multivariate adaptive regression splines (MARS) models. In all cases the PM_10_ values were calculated in two ways: firstly, using the concentration of the six pollutants available as input variables and afterwards employing only four: SO_2_, NO, NO_2_ and PM_10_. The main reason why new models using only four variables of the six available are also trained and validated is that many meteorological stations, including some pertaining to the net of Air Quality Monitoring of the Government of the Principality of Asturias are only able to measure these four variables. In other words, the use of only the aforementioned four variables will allow us to compare the model performance according to the input variables employed and will serve as a reference for future studies. Please note that what was said before relates to all the models of the present research except for ARIMA, where only concentration of PM_10_ are employed for the forecasting. In all cases, for continuous variables minimum, mean, maximum and standard deviation were calculated.

Forecasts are performed from 1 to 6 months in advance. The reason why it might be of interest to perform forecasts 6 months in advance is two-fold. On the one hand, high PM_10_ concentrations have adverse effects on human health and on the other, having such a forecast would be helpful in order to take measurements that would make it possible to comply with European air quality standards. According to the results obtained, the best forecast of PM_10_ concentration 1 month ahead is obtained by the SVMR model calculated with six variables. In the case of the forecast 6 months ahead the results of the MLP with six variables are slightly better. In other words, in the short-term the best forecasts are given by SVMR but in the long-term it is outperformed by MLP.

### Autoregressive integrated moving average (ARIMA)

ARIMA models can be considered as being an extension of ARMA (autoregressive moving average) known for their ability to provide a parsimonious description of a stationary stochastic process^32^. ARMA models are composed of two polynomial terms, one for autoregression (AR) and another for moving average (MA). Given a time series of data $X_{t}$, the ARMA model can be expressed as:

$$X_{t} = c + \varepsilon_{t} + \mathop \sum \limits_{i = 1}^{p} \varphi_{i} X_{t - i} + \mathop \sum \limits_{i = 1}^{q} \sigma_{i} \varepsilon_{t - i}$$

where $c$ is a constant, $\varepsilon_{t}$ are white noise error terms, $\sum\nolimits_{i = 1}^{P} {\varphi_{i} X_{t - i} }$ is the autoregressive addend where $\varphi_{i}$ are parameters and $X_{t - i}$ is the value of variable $X$ in time $t - i$. $\sum\nolimits_{i = 1}^{qq} {\sigma_{i} \varepsilon_{t - i} }$ is the moving-average addend where $\sigma_{i}$ are the parameters of the model.

ARIMA models are appropriate for those observation sets that are not necessarily generated by a time series, as is the case of the present problem. They considerably improve the empirical description of non-stationary time series^29^. A stochastic process can be characterized as an ARIMA model if the d-th difference of $X_{t}$, constitutes an ARMA stationary and invertible process of $p$, $q$ orders.

In this case, $p$ represents the order of the autoregressive part of the model, $q$ is the order of the weighted moving average and another parameter called $d$ represents the number of differencing required to reach stationarity^33^. If the differencing operator is denoted by $\nabla$, the general ARIMA equation can be written as follows^30^:

$$\emptyset_{p} \left( B \right)\nabla^{d} \left( {X_{t} - L} \right) = \theta_{q} \left( B \right)_{{\varepsilon_{i} }}$$

where $\emptyset_{p} \left( B \right)$ and $\theta_{q} \left( B \right)$ are the autoregressive polynomials of weighted moving averages and ${\upvarepsilon }_{{\text{i}}}$ is the model perturbation.

$$\begin{aligned} & \emptyset_{p} \left( B \right) = 1 - \emptyset_{1} B - \emptyset_{2} B^{2} - \cdots - \emptyset_{p} B^{p} \\ & \theta_{q} \left( B \right) = 1 - \theta_{1} B - \theta_{2} B^{2} - \cdots - B_{q} B^{q} \\ \end{aligned}$$

A more in-depth explanation of ARIMA models goes beyond the scope of this research and can be found elsewhere^34^. All the models employed in the present research were calculated with the help of the statistical software R^35^. ARIMA models were calculated with the help of the series library^36^.

### Vector autoregressive moving-average (VARMA)

The Vector autoregression Moving-Average (VARMA) method models the next step in each time series using an ARMA model. In other words, it can be considered the generalization of ARMA to multivariate time series. This kind of model makes it possible to compute a set of time series at the same time, obtaining their within-correlations and cross-correlations^32^. For these models calculus was performed with the help of the MTS library^37^.

If a k-dimensional time series is represented by $z_{t}$, the vector autoregressive moving-average VARMA $\left( {p,q} \right)$ process can be expressed as:

$$\phi \left( B \right)z_{t} = \phi_{0} + \theta \left( B \right)a_{t}$$

where $\phi_{0}$ is a constant vector

$$\begin{aligned} & \phi \left( B \right) = I_{k} - \mathop \sum \limits_{t = 1}^{p} \phi_{t} B_{t} \\ & \theta \left( B \right) = I_{k} - \mathop \sum \limits_{t = 1}^{q} \theta_{t} B_{t} \\ \end{aligned}$$

are two matrix polynomials and $a_{t}$ is a sequence of independent and identically-distributed random vectors with mean zero and positive-definitive covariance matrix $\sum_{a}$.

A general VARMA $\left( {p,q} \right)$ model is represented as follows^37^:

$$z_{t} = \phi_{0} + \mathop \sum \limits_{t = 1}^{p} \phi_{i} z_{t - 1} + a_{t} - \mathop \sum \limits_{t = 1}^{q} \theta_{i} a_{t - i}$$

In this equation $p$ and $q$ are nonnegative integers, $\phi_{0}$ is a vector of constants, $\phi_{i}$ and $\theta_{j}$ are two constant matrix and $\left\{ {a_{t} } \right\}$ is a sequence of independent and identically-distributed random vectors with mean zero and positive definite covariance matrix.

According to Tsay and Wood^37^, the VARMA model expressed in the previous equation can be rewritten in a more convenient way as follows:

$$z_{t} = \phi_{0} + \mathop \sum \limits_{t = 1}^{p} \phi_{i} z_{t - 1} + Lb_{t} - \mathop \sum \limits_{t = 1}^{q} \theta_{j}^{*} b_{t - j}$$

where $\theta_{j}^{*} = \theta_{j} L$ where $L$ is a lower triangular matrix with 1 being the diagonal elements. The determination of $p$ and $q$ values was performed following a methodology suggested in previous research^38^. Akaike information criterion^39^ (AIC) and Schwarz information criterion^40^ (SIC) were employed to balance the improvement in the value of the log-likelihood function with the loss of degrees of the freedom which results from increasing the lag order of a time series model. With the help of both the maximum $p$ *and* $q$ values were calculated. All those models with $p$ *and* $q$ values less or equal to then were calculated and finally, those with the best RMSE were presented in this paper.

### Multilayer perceptron neural networks (MLP)

One of the first bio-inspired machine learning models was the one-layer perceptron. This kind of network was proposed by Rosemblatt^41^ as a possible modelization of the neuron of the human brain. The rule of the perceptron adaption consists of a supervised iterative method that modifies the neuron weights. The multilayer perceptron is useful as a way in which to modelize a function. In a neural network the outcome is modelled by an intermediary data set of unobservable variables called hidden variables, which are linear combinations of the original predictors. However, this linear combination is typically transformed by a nonlinear function.

Kolmogorov^42^ demonstrated that a two-layer network (one hidden layer and one output layer), with a non-linear differentiable activation function is able to approach any “soft” mapping if the number of neurons in the hidden layer is high enough. If a two-layer network like the one employed in the present research is considered, the operations for a system with $p$ input variables, one output variable and $q$ neurons in the hidden layer can be expressed as:

$$y\left( n \right) = \sigma \left( {w^{y} \cdot \varphi \left( {w^{h} \cdot x\left( n \right)} \right)} \right)$$

where $y\left( n \right)$ and $x\left( n \right)$ are the output and input of the net; $\sigma$ is the activation function of the output layer; $\varphi$ is the activation function of the hidden layer; $w^{y}$ and $w^{h}$ are the weights matrix for the output and hidden layer respectively.

One main requirement in order to make possible the MLP training^43^ is that $\sigma$ and $\varphi$ be continuously-differentiable functions. Training is performed with the backpropagation method, which is a recursive application of the gradient descent method. For the purposes of this research, the neural network models were trained and validated with the help of the library neuralnet^44^. The activation function employed is the logistic function. A more in-depth explanation of the foundations of neural networks may be found elsewhere^45^.

### Support vector machines as regressor (SVMR)

Support Vector Machines were introduced by the work of Vapnik^46^. Although they were created by binary classification, nowadays they are used for different kinds of problems. Those employed for regression problems are called SVMR^29^.

Let a training data set $S = \left\{ {\left( {x_{1} ,x_{2} } \right), \ldots \left( {x_{n} ,y_{n} } \right)} \right\}$, where $x_{i} \in \Re^{d}$ and $y_{i} \in \Re$ the regression task involves finding those parameters $w = \left( {w_{1} , \ldots ,w_{d} } \right)$ that make it possible to find the following lineal function^27^:

$$f\left( x \right) = w_{1} x_{1} + \cdots + w_{d} x_{d} + b$$

As in practice it is not possible to find these parameters with a prediction error equal to zero, a concept called soft margin is employed. For this, variable $\xi_{i}$ is employed and the equation is written as follows:

$$\min \frac{1}{2}w,w + c\mathop \sum \limits_{i = 1}^{n} \left( {\xi_{i}^{ + } + \xi_{i}^{ - } } \right)$$

Please note that $\xi_{i}^{ + } > 0$ when the forecast of the model $f\left( {x_{i} } \right)$ is larger than its real value $y_{i}$ and $\xi_{i}^{ + } < 0$ in other cases.

With the help of the lagrangian function and the Karush–Kuhn–Tucker conditions, the problem can be expressed as follows:

$$f\left( x \right) = \mathop \sum \limits_{i = 1}^{n} \left( {\alpha_{i}^{ - } - \alpha_{i}^{ + } } \right)x,x_{0} + b^{*}$$

where

$$\begin{aligned} & \alpha_{i}^{ + } = C - \beta_{i}^{ + } \\ & \alpha_{i}^{ - } = C - \beta_{i}^{ - } \\ & b^{*} = y_{i} - w^{*} ,x_{i} \pm \varepsilon \\ \end{aligned}$$

In those cases where data cannot be adjusted with the help of a linear function, kernels are employed^47^. Kernels transform data into a new space called characteristics space.

The regressor associated to the lineal function in the new space is as follows:

$$f\left( x \right) = \mathop \sum \limits_{i = 1}^{n} \left( {\alpha_{i}^{ - } - \alpha_{i}^{ + } } \right)K\left( {x,x_{i} } \right)$$

please note that $b^{*}$ is not included in the function as it can be included as a constant inside the kernel. The kind of kernel function to be employed depends on the problem to be solved. For example, the radial basis function has been shown to be very effective, but in those cases where the data set comes from a linear regression, the linear kernel function obtains better results^48^. The SVM as regressor models have been implemented with the functionalities of the library e1071^49^. A good explanation of the use of SVM as regressor can be found in the work of Drucker et al.^50^.

### Multivariate adaptive regression splines (MARS)

MARS is a non-parametric modelling method driven by the following equation^51^:

$$y_{t} = f\left( {x_{t} } \right) = \beta_{0} + \mathop \sum \limits_{i = 1}^{k} \beta_{i} \cdot B\left( {x_{it} } \right)$$

where $y_{t}$ is the output variable for each time $t$ and $\beta_{i}$ are the model parameters for the different $x_{it}$. $\beta_{0}$ is the intercept and $B$ represents the model basis functions.

One of the main characteristics of the MARS models is that they do not make use of any a priori hypothesis concerning the relationships among the variables^52^. The basis functions are defined as follows:

$$\begin{aligned} & B^{ - } = \left\{ {\begin{array}{*{20}l} {\left( {t - x} \right)^{q} } \hfill & \quad {if \;x < t} \hfill \\ 0 \hfill & \quad {otherwise} \hfill \\ \end{array} } \right. \\ & B^{ + } = \left\{ {\begin{array}{*{20}l} {\left( {t - x} \right)^{q} } \hfill & \quad {if\; x \ge t} \hfill \\ 0 \hfill & \quad {otherwise} \hfill \\ \end{array} } \right. \\ \end{aligned}$$

$q$ is the power of the basis function as is always a value either equal o larger than zero. In order to adjust a MARS model and decide which basis functions are to be included, MARS makes use of the generalized cross validation (GCV). This represents the root mean squared error divided by a penalty parameter that is defined by the model complexity^53^. Its equation is as follows:

$$C\left( M \right) = M + 1 + d \cdot M$$

where $M$ represents the number of basis functions in the equation and $d$ is a penalty parameter for each base function included in the model. For this research, a value of 2 has been assigned to such a parameter, while the maximum number of tracer interaction type base functions is restricted to 3. The MARS models employed in this research are based on those programmed in the library earth^54^. A complete explanation of MARS models can be found in the original work of Friedman^51^. Also, an easy-to-read introduction to this methodology can be found in the works of Put et al.^55^.

## Results and discussion

Table 2 shows the Pearson’s correlation coefficients of all the variables in the study. The largest correlation coefficient in absolute value corresponds to variables NO and NO_2_ with 0.8626, followed by NO and O_3_ with − 0.7593 (inverse relationship) and SO_2_ and NO_2_ and SO_2_ and NO with 0.7160 and 0.7090 respectively. Correlation coefficients of variables SO_2_, NO, NO_2_ and O_3_ with PM_10_ can be considered in absolute value terms as moderate as they range from 0.4320 (CO and PM_10_) to 0.5251 (NO_2_ and PM_10_).

**Table 2:** Pearson’s correlation coefficients of the variables of the study.

	NO	NO_2_	CO	O_3_	PM_10_
SO_2_	0.7090	0.7160	0.6503	− 0.5483	0.4923
NO		0.8626	0.6587	− 0.7593	0.5068
NO_2_			0.6755	− 0.5475	0.5251
CO				− 0.4823	0.4320
O_3_					− 0.4663

Table 3 shows the results of the ARIMA model using the previous values of PM_10_ as the input variable. Tables 4, 5, 6, 7 and 8 show the results obtained using the different models of four (SO_2_, NO, NO_2_ and PM_10_) and six variables (SO_2_, NO, NO_2_, CO, O_3_ and PM_10_) employed in the present research. In all cases, the results are presented in the same way. The first line represents the forecast performed using information from January 2010 to December 2017 as training values. This forecast is performed for the following 6 months. The second line shows the forecast performed using information from January 2010 to January 2018 and the forecasts from February 2018 (1 month ahead) to June 2018 (5 months ahead) as training values. For all the cases, and in order to make an easy comparison of real values with forecasting, root mean squared errors (RMSE) forecasting values from 1 to 6 months ahead and 1 month ahead for all models are presented in Table 9. In the case of the ARIMA model (Table 3), the one that only makes use of past PM_10_ concentrations in order to predict their future values, the RMSE obtained for forecasts performed 1 month ahead was 6.3163 while the RMSE for forecast performed from 1 to 6 months ahead, the RMSE value was 7.6312. Please note that when we speak about the RMSE obtained for a forecast performed 1 month ahead, we refer to the values that are in the diagonal of the table (in the case of Table 3: 22.2217, 32.0564, 19.7957, 22.9000, 34.6428 and 29.6487) as they are the ones calculated 1 month ahead. Regarding the forecast from 1 to 6 months ahead, we compare real values with the forecast of the first row of the table from January 2018 to June 2018 (in the case of Table 3: 22.2217, 31.5194, 19.8269, 20.1082, 37.0095 and 31.8833). Please note that the real monthly averaged values from January to June 2018 were 29, 27, 26, 31, 29 and 24 respectively. These values are included in Tables 3, 4, 5, 6, 7 and 8 make comparisons more direct.

**Table 3:** Port of Gijón. Results of the ARIMA models using variable PM_10_.

	Jan-18	Feb-18	Mar-18	Apr-18	May-18	Jun-18
	22.2217	31.5194	19.8269	20.1082	37.0095	31.8833
		32.0564	21.4559	22.9140	32.8949	29.8194
			19.7957	23.4279	34.4069	30.4898
				22.9000	33.2961	29.6945
					34.6428	29.3402
						29.6487
Avg	29	27	26	31	29	24

**Table 4:** Port of Gijón. Results of the VARMA models using variables SO_2_, NO, NO_2_ and PM_10_.

p	q	Jan-18	Feb-18	Mar-18	Apr-18	May-18	Jun-18
4	2	39.6108	42.0017	21.0807	39.9202	21.7535	40.9830
4	2		43.1236	24.5948	39.9053	22.4729	41.6383
4	2			21.4770	40.4344	23.8317	34.4105
4	2				40.8564	24.0001	35.0403
4	2					21.8562	33.3678
4	2						32.4333
4	1	40.4407	42.6933	21.5210	40.0030	22.7195	41.5106
4	1		43.8059	24.7208	40.2494	23.4349	41.8195
4	1			21.9345	41.2505	23.9012	34.6527
4	1				41.7434	24.9623	35.1930
4	1					21.9919	33.8758
4	1						32.5900
2	1	40.1493	43.0113	21.3623	39.8731	23.0342	41.7924
2	1		43.9369	25.4061	40.0493	23.9055	41.4342
2	1			22.2033	41.6250	24.4226	34.7485
2	1				41.8263	24.8753	34.8986
2	1					21.7138	34.4839
2	1						32.9979
1	2	39.6597	43.3128	20.8822	39.8591	22.3459	41.5172
1	2		44.7840	25.2376	40.2805	24.0309	40.5961
1	2			21.5377	41.7663	24.4224	34.6140
1	2				41.8746	25.0023	34.8847
1	2					21.5321	34.5682
1	2						32.5428
Avg		29	27	26	31	29	24

**Table 5:** Port of Gijón. Results of the VARMA models using variables SO_2_, NO, NO_2_, CO, O_3_ and PM_10_.

p	q	Jan-18	Feb-18	Mar-18	Apr-18	May-18	Jun-18
4	2	37.8290	44.0486	25.6528	40.2896	25.9201	39.4256
4	2		42.8897	29.4979	36.6604	29.4432	39.6890
4	2			24.6193	36.6633	30.2776	31.0831
4	2				37.7013	29.0341	33.1441
4	2					27.4516	33.9081
4	2						31.8559
4	1	38.7335	43.5777	25.5632	40.6248	26.2742	40.3105
4	1		43.6040	28.9298	37.4593	28.6250	39.3706
4	1			24.2618	37.2412	30.0657	31.7601
4	1				38.8584	28.0238	33.7027
4	1					27.9027	35.1452
4	1						32.9623
2	1	39.0075	44.2329	24.5744	41.0882	26.1770	40.7826
2	1		44.0753	28.3803	39.1346	29.0462	38.8954
2	1			24.7798	37.6661	28.4491	32.0509
2	1				39.2261	27.1545	34.1648
2	1					26.8998	34.9791
2	1						32.8426
1	2	39.6361	44.3558	25.1907	41.3853	25.3394	41.8425
1	2		43.5959	27.5150	39.5201	27.3642	39.6072
1	2			24.0832	39.2072	28.1761	33.7224
1	2				40.5151	26.5538	34.6911
1	2					27.1467	34.9188
1	2						33.0177
Avg		29	27	26	31	29	24

**Table 6:** Port of Gijón. Results of the MLP models with variables SO_2_, NO, NO_2_ and PM_10_ and with variables SO_2_, NO, NO_2_, CO, O_3_ and PM_10_.

	Jan-18	Feb-18	Mar-18	Apr-18	May-18	Jun-18
**Model with variables SO**_**2**_**, NO, NO**_**2**_** and PM**_**10**_						
	22.8982	28.3153	26.0853	20.4793	35.1029	30.9771
		30.2043	26.3742	23.2469	30.1220	31.0470
			25.1822	24.2686	32.2976	29.8388
				25.4043	31.2319	27.8622
					33.6755	27.8836
						29.1743
**Model with variables SO**_**2**_**, NO, NO**_**2**_** CO, O3 and PM**_**10**_						
	23.9208	29.9514	21.0074	22.0775	34.2918	31.4352
		30.3128	19.3318	24.6153	30.3982	30.5587
			24.2857	25.6399	31.9302	29.8496
				26.3989	30.9463	29.5339
					33.3901	29.3019
						29.7334
Avg	29	27	26	31	29	24

**Table 7:** Port of Gijón. Results of the SVMR models with variables SO_2_, NO, NO_2_ and PM_10_ and with variables SO_2_, NO, NO_2_, CO, O_3_ and PM_10_.

	Jan-18	Feb-18	Mar-18	Apr-18	May-18	Jun-18
**Model with variables SO**_**2**_**, NO, NO**_**2**_** and PM**_**10**_						
	22.5224	29.4214	21.7977	21.7465	35.8175	29.2032
		31.2132	19.4056	23.6688	32.2857	28.5922
			24.9580	25.5375	32.5812	29.4920
				26.0547	34.0507	28.3719
					34.4723	28.9101
						29.5071
**Model with variables SO**_**2**_**, NO, NO**_**2**_** CO, O3 and PM**_**10**_						
	23.6383	30.0879	21.4260	21.5572	34.4579	30.7213
		30.9299	19.7200	24.4384	32.0464	29.6021
			25.1539	25.5781	33.4582	30.0453
				26.6899	32.6893	29.9452
					32.9072	28.2090
						29.5126
Avg	29	27	26	31	29	24

**Table 8:** Port of Gijón. Results of the MARS models with variables SO_2_, NO, NO_2_ and PM_10_ and with variables SO_2_, NO, NO_2_, CO, O_3_ and PM_10_.

	Jan-18	Feb-18	Mar-18	Apr-18	May-18	Jun-18
**Model with variables SO**_**2**_**, NO, NO**_**2**_** and PM**_**10**_						
	31.7247	26.2609	27.9016	21.0456	21.7012	41.1329
		25.9874	28.0799	22.6011	22.2278	39.4284
			29.0392	24.4142	23.5083	39.5599
				24.4118	23.5069	39.5599
					23.4797	39.5665
						41.8199
**Model with variables SO**_**2**_**, NO, NO**_**2**_** CO, O3 and PM**_**10**_						
	29.3314	25.8768	32.2319	30.7817	27.1559	39.4833
		25.8768	31.3865	29.9750	26.4461	39.4833
			31.4188	30.0142	26.5028	39.4833
				30.7211	27.1826	39.4833
					27.0815	39.4833
						39.4833
Avg	29	27	26	31	29	24

**Table 9:** RMSE values 1 and up to 6 months ahead of all the models employed in the present study.

Model and variables number	RMSE	
	One month ahead	Up to 6 months ahead
ARIMA	6.3162	7.6312
VARMA (p = 4 q = 2) 4 variables	10.1021	11.4189
VARMA (p = 4 q = 1) 4 variables	10.5529	11.7214
VARMA (p = 2 q = 1) 4 variables	10.6211	11.7832
VARMA (p = 1 q = 2) 4 variables	10.7767	11.8007
VARMA (p = 4 q = 2) 6 variables	8.5767	10.8202
VARMA (p = 4 q = 1) 6 variables	9.2802	11.0743
VARMA (p = 2 q = 1) 6 variables	9.5173	11.4786
VARMA (p = 1 q = 2) 6 variables	9.7252	11.9347
MLP 4 variables	4.6209	6.2661
MLP 6 variables	4.3402	6.0873
SVMR 4 variables	4.9249	6.1191
SVMR 6 variables	4.2649	6.1010
MARS 4 variables	8.2575	8.7319
MARS 6 variables	6.7605	6.8725

The RMSE values achieved 1 and up to 6 months ahead for all the models trained in the present research are shown in Table 9. For forecasting 1 month ahead, the best results are obtained for the six variables of SVMR and MLP models, followed by the same models including only four variables. These results give us the idea that all the variables included in the study have a certain relevance in terms of performing an accurate PM_10_ prediction. After the MLP and SVMR models, according to RMSE values the next best in forecasting 1 month ahead is the ARIMA model, the only one that makes exclusive use of past PM_10_ values in order to forecast future concentrations. The ARIMA model is followed by MARS with six and four variables, while VARMA are the models that give the worst performance.

In the case of a forecast of up to 6 months ahead, the best performance according to RMSE value is also achieved by 6 variables MLP and SVMR models followed by the same models using only four variables. A remarkable change when compared with the forecast 1 month ahead is that the MARS model that includes 6 variables performs better than the ARIMA model. Finally, and as also happened with the forecasts 1 month ahead, the worst performance was shown by the VARMA models.

From our point of view, a remarkable fact is that the model performance in terms of RMSE in both 1- and 6-month ahead models is not only linked to the number of variables considered in it, but also to the kind of model selected. In other words, it is possible to find a model of only one variable (ARIMA) that performs better than others that include six variables in both 1- and 6-month ahead predictions (VARMA). Finally, the importance of a variable is very easy to assess with the help of a MARS model. The importance order found for the prediction of PM_10_ was as follows: PM_10_ value in the previous moments, followed by the previous measurements of CO, NO, O_3_, SO_2_ and NO_2_.

The main limitation of this study is that although original data is taken each 15 min, forecasts are performed for average monthly values. The reason why average monthly values were forecasted is that the results obtained by the authors when daily or hourly forecasts were performed were not as stable as the average monthly values. This is due to the influence of the port traffic in the pollution area, which does not follow a fixed cycle like urban traffic. Another limitation to be overcome in future studies is that in order to improve the results obtained it would be of interest to introduce some meteorological variables such as temperature, humidity, pressure, sun radiation, rainfall and wind speed and direction.

## Conclusions

The results obtained in this research allow us to say that it is possible to predict PM_10_ concentration with the help of the value of this variable and the concentration of other pollutants by means of statistical and machine learning models. Also, another interesting issue is that as had already been found in previous studies,^56^ the use of the concentration of other pollutants helps to obtain a more accurate prediction. In fact, the most accurate results were obtained for two kind of machine learning models, SVMR and MLP, when they made use of the values of the six available variables. The results obtained show how regression-based models like SVMR, MARS and MLP outperform univariate and multivariate time series-based models (ARIMA and VARMA). According to the findings of this paper and other previous ones^29^, this is because the short-term relationships among pollutants are stronger than temporal relationships of PM_10_ concentration values with itself and with other variables. In other words, although it is possible to find certain seasonal patterns in monthly average pollutant values, the relationship of PM_10_ with the concentration of other pollutants is more important than the seasonal pattern.

Finally, this research affords the reader the opportunity to compare different machine learning and time series methodologies applied to the same data set to establish whether they are useful for PM_10_ concentration forecasting. If the average monthly values of PM_10_ from January to June 2018 are compared with those corresponding to the same months of the previous year, the RMSE result is 6.8557. This means that in forecasts 1 month ahead, MLP and SVM models of four and six variables and MARS of six variables outperform it. When forecasts are performed 6 months ahead MLP models of four and six variables and SVM of six variables outperform it. Although the proposed methodologies do not always outperform the mere use of the average values of PM_10_ concentrations of the same months of the previous year, they are a useful complementary tool for planning and taking decisions in advance.
